# JUNO Project: Deployment and Validation of a Low-Cost Cloud-Based Robotic Platform for Reliable Smart Navigation and Natural Interaction with Humans in an Elderly Institution

**DOI:** 10.3390/s23010483

**Published:** 2023-01-02

**Authors:** Nieves Pavón-Pulido, Jesús Damián Blasco-García, Juan Antonio López-Riquelme, Jorge Feliu-Batlle, Roberto Oterino-Bono, María Trinidad Herrero

**Affiliations:** 1 Automation, Electrical Engineering and Electronic Technology Department, Industrial Engineering Technical School, Technical University of Cartagena, 30202 Cartagena, Spain; 2 Clinical and Experimental Neuroscience (NiCE), Institute for Aging Research, Biomedical Institute for Bio-Health Research of Murcia (IMIB-Arrixaca), School of Medicine, University of Murcia, Campus Mare Nostrum, 30120 Murcia, Spain

**Keywords:** smart assistant robots, human–robot natural interaction, autonomous navigation, digital transformation of health systems

## Abstract

This paper describes the main results of the JUNO project, a proof of concept developed in the Region of Murcia in Spain, where a smart assistant robot with capabilities for smart navigation and natural human interaction has been developed and deployed, and it is being validated in an elderly institution with real elderly users. The robot is focused on helping people carry out cognitive stimulation exercises and other entertainment activities since it can detect and recognize people, safely navigate through the residence, and acquire information about attention while users are doing the mentioned exercises. All the information could be shared through the Cloud, if needed, and health professionals, caregivers and relatives could access such information by considering the highest standards of privacy required in these environments. Several tests have been performed to validate the system, which combines classic techniques and new Deep Learning-based methods to carry out the requested tasks, including semantic navigation, face detection and recognition, speech to text and text to speech translation, and natural language processing, working both in a local and Cloud-based environment, obtaining an economically affordable system. The paper also discusses the limitations of the platform and proposes several solutions to the detected drawbacks in this kind of complex environment, where the fragility of users should be also considered.

## 1. Introduction

The COVID’19 pandemics have demonstrated that health systems could be highly stressed, and the attention of vulnerable people is difficult, not only in this kind of situations, but in general, since population aging is happening throughout the world [1] and life expectancy is also growing, both in developed and developing countries.

The fact of living longer lives is actually good news; however, elderly people’s quality of life is often unsatisfactory since certain diseases and emotional events affect them more virulently to higher ages [1]. Current social interactions, often based on the use of technology, are also obstacles for elderly people that push them to live in solitude, even if they stay in elderly homes. This situation worsened in the pandemic age [2]. Although, in the post-pandemic age, rules of isolation for people have been relaxed or even eliminated in many countries, comorbidity and immunology system decline keep on reducing the possibility of living a normal life in some vulnerable groups exposed to severe forms of COVID, which clearly could affect non-vaccinated or susceptible vaccinated people, especially those older than 65 and with previous pathologies (multimorbidity).

Note that during some periods, mainly with a high COVID incidence, medical telephonic attention was the main or even the only method for remote health attention and communication with relatives, most of them insufficient for many users. This fact has demonstrated that there exists a lack of both an appropriate digitization of health systems and the availability of digital tools, ready to be used by elderly people with low or zero knowledge about how to use technology, making difficult to enjoy its advantages. 

On the other hand, personalized treatment and tracking of psychological and emotional diseases are difficult in elderly institutions, often with a lack of staff, so patients’ mood and anxiety disorders often worsen, especially in those with cognitive decline or in their prodromes. These circumstances deteriorate the general health state and users are exposed to a worse quality of life.

However, Information and Communication Technology (ICT) can offer interesting solutions for facing and dealing with this social-health care problem, which not only actually affects elderly patients, but their caregivers and relatives [3]. Such solutions go through health care digitization by considering all the disruptive technologies that have emerged in recent years, such as Service Robotics, Cloud and Edge Computing, Machine Learning (ML) and Deep Learning (DL), Natural User Interfaces (NUIs) and Internet of Things (IoT), among others, which are successfully penetrating in many sectors and social contexts. This is the reason why, in the last decade, some research European programs, such as the AAL (Ambient Assisted Living), program [4,5,6,7,8,9,10,11,12,13], have encouraged the scientific community to develop and deploy new digital solutions for improving the lifestyle of senior citizens, with the aim of increasing their autonomy and reducing the risk of being institutionalized.

Many of the solutions found in the state-of-the-art, or in past and on-going projects are focused on providing technology for senior citizens who are capable of using it. However, others propose the use of social robots and environments with IoT-based monitoring that uses ML-based techniques to analyze the behavior of users and propose activities for combating and slowing down the cognitive decline progression. The project engAGE is an example of this approach [5]. In this case, “the primary end-users are older adults over 65 with mild cognitive decline, living independently and supported by caregivers, needing care assistance to self-manage and support their cognitive function”. This project started on 1 December 2021, but there are not enough results to compare to yet, although their researchers expect, according to [5], to provide three main services (built around a social robot) for “monitoring daily life activities, health state and wellbeing, assessing cognitive state and potential decline by leveraging machine learning algorithms and offering personalized cognitive function support and coaching”. One of the main limitations of this system is the use of the robot Pepper, since its economic cost is very high for elderly people with low resources. On the other hand, the project is aimed at people living at home, not for many users living together in an elderly institution.

Another project in the AAL program, ReMember-Me, is considered “an effort to develop a solution to detect and prevent cognitive decline early on” [6]. In this case, the solution also pretends to include a robot and provide screen games and sensors to capture data about the person’s status. A monitoring platform would also be included to enable communication between older adults and their caregivers, and a social platform would make social interaction easier. This project started in April 2020, with a duration of 36 months, and several results have been obtained as publications, mainly related to daily activity identification [14,15] or using wearable sensors to detect human behavior; however, only one publication is focused on describing how robots could be introduced in this context [16].

The project AgeWell [7,17] is another example of a solution to provide “an avatar on a mobile phone and robot (physically embodied device) based virtual coach for older employees supporting them through their transformation to retirement and beyond.” However, such a solution is not specifically centered on elderly people with a certain degree of vulnerability in an elderly institution.

The project eWare [8] “is focused on improving the lifestyle of people with dementia and their caregivers”, taking into account that “several technologies and services have been developed to support the care for people with dementia”. Therefore, researchers in this project try to integrate lifestyle monitoring (through Sensara motion and open/close sensors), with the social robotic technology of Tinybots. In this case, the “robot” is really a device that monitors the habits and activities of people with dementia, but it is not a mobile robot.

The CAMI project offers an AAL-based solution for providing services related to health and home management and well-being, and it integrates a telepresence robot, but the system is also aimed at elderly people with good abilities and living at home with informal caregivers [9].

The projects ExCITE [11], ALIAS [12] and DOMEO [13] are similar to CAMI since they integrate mobile robot prototypes mainly for telepresence but, in general, the economic cost of building such platforms is currently high.

Referred solutions are mainly designed to allow elderly people to stay at home with informal caregivers; nevertheless, in many cases, due to several social factors, elderly people need to stay in elderly homes during some periods of time, or permanently. As aforementioned, several solutions are focused on providing AAL-based environments, including the use of assistive robots [18,19,20]. Many of these solutions require the installation of more or less complex systems in buildings, such as cameras or sensors in furniture, and although the idea of using this kind of device is interesting, since home automation is successfully evolving quickly from the commercial point of view, the installation procedure is often tedious and privacy issues are not always satisfactorily treated. Moreover, this kind of systems, if conceived for helping elderly people, should be designed by considering the lack of knowledge about ICT among many of them [21,22], because if these restrictions are not taken into account, solutions will not be accepted by such users, making a suitable transference to market difficult. Therefore, including robots capable of getting closer users and offering them a natural interface for sharing information should be the key for developing efficient digital assistance systems in the context of elderly institutions.

It should be noted that, in recent years, many contributions have been individually made in different fields, such as Robotics, Artificial Intelligence (AI), Speech to Text (STT) and Text to Speech (TTS) systems, Natural Language Processing (NLP), Artificial Vision and Cloud Computing [23]. In particular, in the area of robot autonomous navigation [24,25,26,27,28,29,30], interesting advances have been made in Simultaneous Mapping and Localization (SLAM) by using metric, topological and semantic paradigms, including multimodal perception. Other interesting results in navigation have been recently obtained, for example, those detailed in [31,32,33,34,35,36]. Human Robot Interaction (HRI) in a natural manner and the concept of Social Robotics are also very active topics of interest. Advances in Cloud Computing and IoT are enabling ICT penetration in many contexts, such as e-Health and telemedicine [37,38,39,40,41,42,43,44,45,46,47,48]. Finally, it is interesting to highlight that Deep Learning offers many advances in many areas, from robot navigation [49] to biomedical applications [50,51].

Although the state-of-the-art reveals that there are numerous research efforts focused on the development of e-Health systems that include service robots for helping people [52,53,54], in the context of elderly homes, it is currently difficult to find solutions near the market related to remote assistance of elderly people, since such solutions should include economically affordable assistant robots capable of working locally and/or connected to the Cloud, with certain ability of understanding the context, performing long-term reliable and safe autonomous navigation and using human–robot natural interaction as much as possible, with user interfaces adapted to potential users with different roles, that is, elderly patients, caregivers and Health professionals in residences.

The work presented in this paper is part of a project developed during the year 2022 as a proof of concept at the Technical University of Cartagena (UPCT) and funded by the Fundación Séneca (an institution in the Region of Murcia, Spain). The final objective of the project is to validate an economically affordable autonomous smart robotic platform named JUNO, deployed in a real elderly institution, and previously tested in a controlled laboratory environment, with characteristics very similar to the final setting. The JUNO robot is designed to help elderly people to improve their emotional and mental health by allowing them to carry out several daily tasks focused on cognitive training through cognitive stimulation exercises.

JUNO’s users are not capable of using typical well-known applications, such as e-mail, video calls or social networks, or even handling the most basic aspects of an Operating System (OS), such as Windows, iOS, or Android. Consequently, the system has been designed to allow the robot to recognize each user who participates in the proof of concept, interacting in a natural manner and navigating both autonomously and guided by using voice commands. The main novelty of the software architecture presented in this paper is not in each particular component but in validating the integration of many existing open-source software modules to build a robust system capable of helping elderly users, with certain cognitive impairment, to follow a program of exercises for cognitive stimulation, without the need of overloading the daily tasks carried out by the residence’s professionals, for example, medical staff or occupational therapists, among others.

In addition, the robot allows the results of exercises to be stored in the Cloud for facilitating professionals to track the program of cognitive rehabilitation.

The outline of the paper is as follows: Section 2 presents the system architecture, describing the main features of the robot from a hardware point of view, since it has been designed as an economically affordable robotic platform in comparison with other commercial robots. Furthermore, the software architecture is also described explaining, in detail, which existing open-source software components, including DL-based solutions, have been integrated by using the Robotic Operating System (ROS) framework. The software architecture allows the JUNO robot to safely navigate through a hybrid map (metric and topological), detecting and recognizing faces, locally understanding spoken commands without the need to use Internet resources or commercial smart speakers, presenting cognitive stimulation exercises to users, and evaluating their degree of attention while they carry out them. Section 3 details the set of tests designed to test JUNO, both in the laboratory and in the real residence, and the obtained results. Finally, in Section 4, a discussion about the advantages and limitations is presented, together with the main future work focused on addressing the main issues related to the detected limitations.

## 2. Materials and Methods

As mentioned in Section 1, the novelty of this work is not the development of individual components for solving problems typically related to autonomous navigation or natural interaction between humans and robots. This work emphasizes the process of combining existing solutions, which have been modified and extended as needed, with the purpose of enhancing the performance of a smart assistant robot deployed in a real environment, interacting with real users with very low or zero knowledge about technology, and with the possibility of suffering some kind of physical and/or cognitive impairment.

This section describes the hardware and software architectures used for JUNO’s implementation and shows the methodology used for obtaining such an integral system by combining and improving, if needed, already tested existing software components designed as open-source modules.

### 2.1. Hardware and Software Design

The main challenge of this work is related to the economic cost, external design of the robot and restrictions in terms of performing safe navigation and human–robot interaction. Therefore, the design is focused on saving economic cost but maintaining a suitable performance, in terms of suitable operation of an assistant robot by using existing ROS-based modules well-known in the state-of-art for navigation, and other components for face detection and recognition, for STT and TTS processes and for NLP, all of them based on existing open-source solutions or free available ML models, properly modified and validated for their integration in the ROS ecosystem.

#### 2.1.1. The Robot JUNO

JUNO has been developed by considering certain aspects of design with the aim of gaining a better acceptance of it in a non-technological context. In fact, JUNO is a wheeled robot, but intended to be similar to a piece of furniture. A neurologist and expert in aging has collaborated in its design, considering aspects related to size, weight, shape, and proportions, among others. The opinions of other professionals, including a psychologist, an occupational therapist, and other members of the residence’s staff have also been considered in defining the behavior of the robot. This collaboration between technicians and health professionals is also novel, since many developments are being carried out without taking into account the hard restrictions related to mobility or the type of interaction with the robot that is possible in elderly residences, mainly where impaired people stay.

Several requirements, which have been taken into account after acquiring information from experts in aging and professionals of elderly institutions, are summarized as follows:

- The height of the robot should be the needed to put the tactile screen in an accessible position for being used by users who could often be in a wheelchair. Thus, the tactile screen stands in a typical TV stand, manually configured in the x, y and z axes.
- The tactile screen includes hidden speakers, and the RGB-D sensor provides microphones, so playing and recording sounds is possible.
- The weight of the robot should be high (around 30 kg), because keeping the balance is very important, since vulnerable users could lean on it, and stability is an essential feature required in this context.
- The robot does not present sharp corners or pieces that stand out of the imaginary cylinder used to ensure collision avoidance. This is very important because elderly people living at the residence can easily suffer injuries, even if they are slightly beaten by the robot due to an unexpected collision.

**Figure 1:**
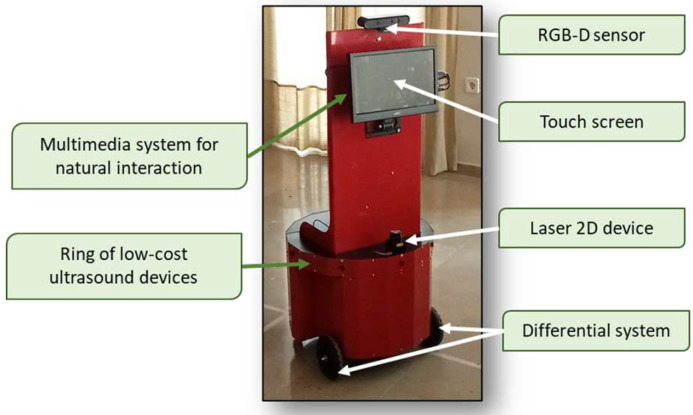
JUNO robotic platform. The radius of the widest part of the robot is 0.25 m and the height is around 1.60 m.

Figure 1 shows the external design of JUNO. Table 1 details the hardware components of JUNO’s equipment.

Note that acquiring economically affordable sensors and actuators is essential, since, as it is pretended to market the robotic platform soon, under an own commercial brand, its economic cost should be as low as possible, without losing the main capabilities that an assistant robot should have. Obviously, the acquisition of low-cost sensors and actuators often affects crucial navigation components, such as those involved in odometry computation based on “dead reckoning.” However, the decrease in the price of Laser 2D devices, such as the marketed by SLAMTEC, for instance, or the more affordable versions of Hokuyo lasers, make the use of localization probabilistic techniques easier. In fact, JUNO uses the *amcl* ROS package for global localization and position tracking, and although a less accurate odometry module has a worse influence on global performance, the *amcl* module has been empirically configured for getting a tradeoff between cost and reliability.

#### 2.1.2. Functionality of JUNO and Software Architecture Overview

**Figure 2:**
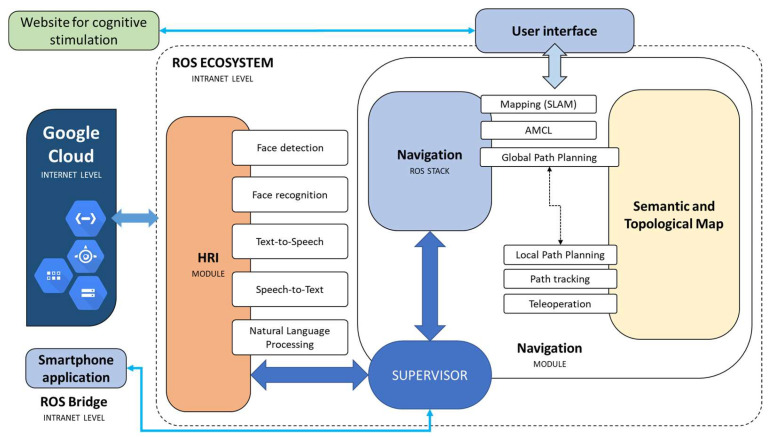
Diagram with all the components included in the whole software architecture.

**Figure 3:**
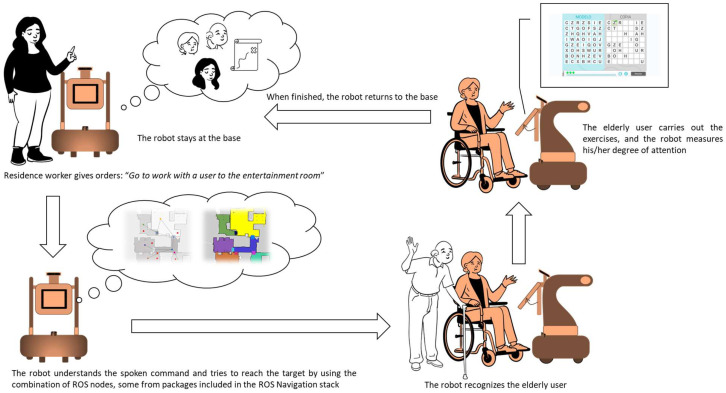
Standard mode of operation of the robot for helping elderly users to do their cognitive stimulation exercises. The robot stays at the base and the arrows represents the flow of tasks that the robot should accomplish from the moment that the residence worker gives an order, until the robot complete the activities and returns to the base.

On the other hand, Figure 2 shows a global diagram of the software architecture that allows JUNO to carry out the following tasks for performing the activities (see Figure 3), specified by the health professionals:

- Being teleoperated by using simple and complex voice commands without the need of using the Internet or smart speakers.
- Changing between teleoperation and autonomous navigation modes by using voice commands, the tactile screen, and a web application capable of interacting with JUNO through the private Intranet.
- Navigating to a specific location in a semantic map, which is superposed over a metric map obtained by using the *gmapping* ROS package. The first map includes a collection of relevant positions from a topological point of view, which are defined during the installation process of the robotic platform. Such locations can be specified by using natural language using an NLP system designed for directly transforming complex navigation orders into ROS-based code that allows velocity commands to be published on a *topic* that exposes typical *Twist* messages defined in the *geometry_msgs* package.
- Finding a specific elderly user by using face detection and recognition techniques and presenting the cognitive stimulation exercises programmed by the therapist. Once the user finishes the exercise, JUNO should return to the base.

### 2.2. Mode of Operation

According to Figure 3, the activities that define the daily behavior of the robot are related to helping the selected elderly patients complete their daily cognitive stimulation exercises.

The robot stays at its base until it is requested to help an elderly user with his/her exercises. Elderly users’ information has been previously uploaded during the system’s installation procedure, together with the metric and semantic maps and the DL models for each person’s face that should be automatically recognized. Furthermore, specific locations in the semantic map are properly labeled, defining different points of interest, such as the base and the place where people carry out their exercises, among others.

A typical graphical user interface allows the residence’s worker, in charge of handling the robot, to select these main options: (i) voice teleoperation; (ii) selecting an elderly user; and (iii) activating the robot for helping the selected user.

If voice teleoperation is active, the robot waits for spoken simple motion commands, such as “Adelante” (go), “derecha” (right), “izquierda” (left) and “atrás” (go back). Note that the spoken interaction is in Spanish. This is useful when it is necessary to help the robot to recover itself from navigation failures.

Elderly people carry out their activities of occupational therapy in a specific place in the residence. Then, cognitive exercises are completed in such a place by following a schedule specified by the health professionals. As the robot knows this location, since it is stored as a relevant one in the semantic map, the residence’s worker selects the user and sends the robot to perform the task of helping him/her. The robot autonomously navigates to the target position, and when it reaches it, it searches for the user by using the face detection and recognition module. Then, it performs the needed maneuvers to stand in front of the elderly user, and he/she carries out his/her session of cognitive exercises. The robot facilitates the start of the session by automating the process of identification through the face recognition solution, and it evaluates the degree of attention paid by the user during the execution of the cognitive exercise by analyzing facial motion. All the results are properly stored locally, and they are synchronized with the Cloud when requested. Finally, when the session is finished, the robot autonomously returns to the base.

### 2.3. Description of the Software Components

Several existing components have been included in the software architecture for giving the robot the following capacities: autonomous navigation, face detection and recognition, STT, TTS and NLP abilities.

#### 2.3.1. Autonomous Navigation Modules Existing in the ROS Framework

The ROS *Navigation* stack has been selected for solving the problem of autonomously navigating through the environment. In particular, the following packages have been used:

- ROS *gmapping* package: It allows the metric map to be generated during the initial installation stage by using the FastSLAM method for performing SLAM (Simultaneous Localization and Mapping) [55]. The obtained map is then saved by using the node *map_saver* found in the ROS *map_server* package.

During this stage, technicians in charge of installing the system at the elderly home teleoperate the robot by using a smartphone application specifically designed for this purpose.

- ROS *amcl* package: It implements the adaptive Monte Carlo localization approach, which uses a particle filter to track the pose of a robot against a known metric map published on a topic that stores *OccupancyGrid* messages defined in the ROS *nav_msgs* package. The *amcl* package’s authors used several algorithms described in [24] to define the odometry sample motion model and the beam range finder model, among others. This involves the need to adjust a wide number of configuration values, such as the number of maximum and minimum particles, or the parameters that define the laser and odometry models, among others [56]. Such configuration has been made empirically after testing the robot in different situations, both in the laboratory and in the final real environment.
- ROS *move_base* package: Once the robot is capable of being localized on the global map, it should reach target locations when requested, in a safe manner. The purpose of the *move_base* package is to provide an implementation of a ROS action that, given a target location in the world, the robot reaches it by following a safe path, autonomously calculated by considering data from corrected odometry (through *amcl*), sensors and the map of the environment defined as a 2D occupancy grid [56]. The *move_base* package supports planners adhering to the *nav_core::BaseGlobalPlanner* and the *nav_core::BaseLocalPlanner* interfaces defined in the ROS *nav_core* package. It maintains two superposed maps over the global map, one for the global planner and another for the local planner, defined as cost maps, which take in data from the 2D laser device and build the new maps by considering the information provided by the global metric map and an inflation radius defined by the developer. Such an inflation radius is exposed as a configurable parameter.

In particular, in this work, only the global planner is used, specifically the *navfn*, which uses the Dijkstra’s algorithm to find a global path with minimum cost between the start and end locations.

- ROS *map_server* package: After the installation procedure, the metric map calculated by *gmapping* is stored. Another map is obtained offline as an occupancy grid, where each cell represents a number assigned to a specific area (defined as a semantic concept, such as a room, a corridor, or another specific region of the free space). Each defined area is additionally represented by a location. The locations are connected by using a graph that defines a topological map superposed over the semantic and metric maps. The metric map is published by using the node *map_server* implemented in the *map_server* package.

#### 2.3.2. Techniques Used in Natural Interaction Software Modules

Elderly person detection is carried out by using an RGB-D sensor (Orbbec Astra Pro model), which is integrated into the ROS framework by using the official ROS driver for Orbbec 3D cameras. In particular, face detection and recognition are carried out by applying the following techniques:

- Face detection module: A face detector that uses the pre-trained Deep Learning OpenCV’s Caffe model has been tested. Such a model is actually part of OpenCV since OpenCV 3.3, and it is based on the Single Shot-Multibox Detector (SSD) [57], which uses the ResNet-10 [58] architecture as its backbone.
- Face recognition: The FaceNet system is used for this purpose since it “directly learns a mapping from face images to a compact Euclidean space where distances directly correspond to a measure of face similarity” [59]. FaceNet has been trained by using hundreds of millions of images. Consequently, the obtained accuracy is more than 99% for face recognition.
- Speech to Text (STT): The Vosk speech recognition toolkit [60,61] has been selected for STT, since it supports a wide number of languages and dialects; it works offline even on lightweight devices; it allows the possibility of reconfiguring the vocabulary for improving the accuracy; and it supports speaker identification if needed, although this feature is not used in this work yet. The chosen language for this work is Spanish and the selected model is a lightweight wideband one appropriated for Android devices and even Raspberry Pi computers.
- Text to Speech (TTS): A combination of the library pyttx3 [62] and talkey [63] (an interface library with multi-language and multi-engine support) has been used to give JUNO the capacity to speak certain simple Spanish phrases. In addition, the web application, which allows elderly users to complete the program of cognitive stimulation, uses its own TTS system. JUNO’s tactile screen includes a pair of speakers to reproduce any kind of sound.
- Natural Language Processing (NLP): The library spaCy (a free solution written in Python) [64] is used to extract relevant information from the output generated by the software module that uses the Vosk STT. Such information is a collection of commands specifically related to the process of navigation and for changing the operation mode of the robot by using Natural Language.

The spaCY solution offers a language-processing pipeline; that is, when it is called, spaCY first tokenizes the text and produces an object named Doc. This object is then processed in several different steps or processing pipeline. Each pipeline component returns the processed Doc, which is passed on to the following processing module. The capabilities of a processing pipeline always depend on the components, their models and how they were trained. The collection of included components in the pipeline is configurable. In particular, in this work, the “lemmatizer” has been used since it allows lemmas or base forms to be assigned. In Spanish, this is especially interesting for detecting actions represented by verbs, independent of tense, number, and person. The syntactical analyzer is implemented by the Matcher class, which allows tokens to be classified according to certain syntactical rules.

Although JUNO currently comprehends a very reduced set of simple commands and certain sentences that describe orders related to motion, spaCY has also been chosen because the process of extending its understanding capabilities in the future would be easier.

- Attention degree analysis: Estimating the degree of attention when a user is carrying out a specific cognitive exercise is implemented by using the MediaPipe solution FaceMesh, which is capable of estimating 468 3D face landmarks in real time, even in lightweight devices [65,66]. It uses an ML-based method to infer the 3D facial surface from RGB images. It consists of a pipeline that combines two real-time deep neural network models working together: a detector acting on the full image and calculating face locations and a 3D face landmark model that operates on the selected locations of the image for predicting the 3D surface by using a regression technique. Once the face mesh is obtained, interesting landmarks are selected to obtain a measurement of the attention degree.

#### 2.3.3. Integration of the Software Modules

The software modules that include the techniques described in Section 2.3.1 and Section 2.3.2 are designed as ROS nodes since the software architecture is designed as a set of fully decoupled components that share information through the ROS ecosystem (see Figure 2).

As mentioned in Section 2.3.1, when the *move_base* package is used, only the global planner is considered. A new local planner has been implemented for this work after testing the main local planners offered by *move_base*, since the performance of such planners does not meet the expectations of the JUNO project. In this section, all of the new contributions made in the context of this work are detailed:

- Local planner: A specific ROS node for explicitly tracking the path generated by the global planner has been implemented. The Pure Pursuit [67] technique is used to follow such a path by adapting the behavior of the robot according to different situations. Thus, if the robot is not aligned with the position to be tracked (located at a given lookahead *L*), it rotates on itself until it aligns the robot and the target position (a threshold of 15 degrees is used for deciding that JUNO is aligned). The value of *L* and the velocity are adaptive, since if the robot is aligned to the target point, linear velocity is incremented, but when it rotates, such velocity decreases. A secondary lookahead L2, defined as an empirically configurable distance (0.75 m in this work), is used to search for positions in front of the robot when it is aligned with the path. If the position at L2 belongs to a different area in the semantic map in comparison to the current position of the robot, the lookahead *L* decreases together with the linear velocity. This improves the behavior of the robot when it traverses narrow spaces, such as doors.

In fact, the main problems of typical local planners implemented with *move_base* offer an unreliable performance in this kind of situation because such behavior depends on the heuristic configuration of a wide range of parameters. Therefore, the solution presented in this work is a novel contribution for avoiding some unexpected motions generated by certain local planners (found in the state-of-the-art) implemented in *move_base*.

To avoid dynamic obstacles, JUNO generates a local map based on a set of semantically labeled polylines, according to the technique used in [52,68]. If there are possible traversable segments compatible with the dimensions of the robot, such obstacles are projected over the global map as occupied cells, and the calculation of a new global path is requested, considering the dynamic obstacle, by simply publishing the goal destination again and allowing the global planner to execute the new plan.

If there are no traversable segments compatible with the robot, the robot is automatically sent to another destination. In this case, the semantic map included a set of positions considered as emergency locations. In the elderly institution, such locations are those where the robot can stay without disturbing elderly people who are moving through the environment. Once the robot reaches one of these locations, it stays there during a configurable period of time. Then, it tries again to reach the initial goal position. After a configurable number of attempts or if it is not possible to reach any emergency position, the robot stops and emits an emergency message to the smartphone application that uses the residence’s worker in charge of the robot.

**Figure 4:**
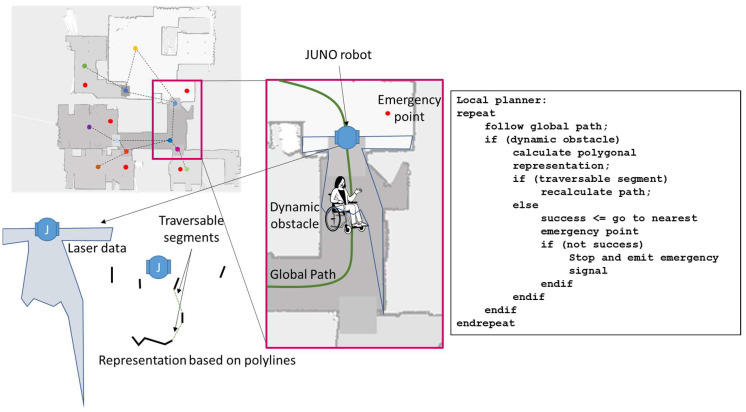
Summarized behavior of the local planner. Representation of the scenario where a dynamic obstacle appears, and it is not possible to find a traversable segment from the polygonal representation. The emergency point is located near the robot; therefore, the algorithm drives the robot to the emergency point. Note that the red points are considered as emergency points in different regions. The rest of points with different colors are representative of each area in the semantic map. Arrows detail the location of the JUNO robot in the map, the laser data and its polygonal representation from the point of view of the robot.

Figure 4 summarizes the main steps of the designed local planner, which improves the performance of the navigation process.

- Supervisor node: This ROS node has been specifically implemented to centralize all the data sent by the rest of the nodes. According to such data, it is possible to change the mode of operation and, thus, send the appropriate velocity command to the robot.
- Low-level JUNO controller: A ROS node for sending velocity commands and estimating the odometry from encoder readings has been implemented. Such a node sends and receives a set of data frames (package of bytes) to and from the firmware executed in a Raspberry Pi Pico, where the low-level PID controller runs. Such firmware has also been fully designed by authors with the purpose of reducing the economic cost of the traction system while maintaining a suitable performance for odometry calculation by using the “dead reckoning” technique.
- Face detection and recognition node: This ROS node integrates the techniques for face detection and recognition described in Section 2.3.2. When the robot reaches a goal position considered a location where an elderly person is waiting to carry out his/her programmed cognitive stimulation exercises, this node is activated, and the robot rotates on itself until the face of the elderly user is detected and recognized. If the process fails, the robot sends a message to the smartphone application mentioned above.

As explained in Section 2.3.2, FaceNet allows faces to be recognized by using a transformation from images to vectors. Then, recognizing the face could be reduced to the comparison of pairs of vectors using, for example, the Euclidian distance or other more complex classifiers.

In this work, during the first installation of the system, for each user who should be recognized, several image cutouts with the face of such a subject are propagated through FaceNet. The vectors obtained as outputs are locally stored and synchronized with the Cloud if requested. These vectors have been used to allow a classifier to be trained for several users’ identification, improving the recognition procedure.

In terms of privacy, the FaceNet method is particularly useful in the context of elderly people’s identity recognition since only the resulting tensor generated by the neural network is saved.

- STT and NLP nodes: A ROS node uses the STT system described in Section 2.3.2 to acquire sound from a microphone and translate sounds into text. Such text is then analyzed by the NLP system, and only if a sentence with sense has been perceived it is sent to a specific topic that stores String messages defined in the *std_msgs* ROS package.
- Attention degree analyzer: It is implemented as a ROS node that publishes, through the corresponding “topic”, the value of the direction of the face. Such direction is calculated using the three-dimensional coordinates of the set of representative points generated by the MediaPipe FaceMesh solution. Specifically, once such coordinates are obtained, the algorithm analyzes the variation of the depth value (z coordinate) of the eyes and mouth, and it determines where the user is looking.
- Smartphone application: It is implemented as a web application that runs under any compatible browser that supports the JavaScript ROS bridge suite. It connects to the ROS ecosystem through a WebSocket; consequently, it is requested that the smartphone is connected to the same intranet as the robot. This application allows the person in charge of the robot to perform an emergency stop, to teleoperate it, if needed, and he/she can receive messages from the robot in special situations when an elderly person is not detected in the target location, or it is impossible for the robot to reach a navigation goal.
- User interface application: As the robot is equipped with a tactile screen, a typical user interface is presented when the robot is working. Such an interface provides options for the residence staff, making the use of JUNO easy. In particular, professionals can select elderly users and send the robot to different locations by simply selecting a place and pushing a button.

Additionally, when the robot has to present the cognitive exercises, it is necessary to connect to an external web platform (*NeuroUP*), which allows elderly users to perform the programmed exercises. Such an application requires the introduction of a login name and a password because certain results obtained after finishing a specific exercise are remotely stored in the private Cloud managed by the company that provides the service.

For impaired people or those with zero knowledge about ICT, introducing this data is very difficult, so a residence’s worker would be required to stay with the robot in this moment. However, this situation could interfere with the daily tasks that such workers should perform.

Each elderly user has a unique identifier associated to his/her identity. This information is encrypted and stored in the computer that controls the robot together with a password and is also saved in the Cloud. 

When the corresponding node detects and recognizes a user, it publishes such an identifier in a ROS topic to which the user interface application is subscribed. Then, such an identifier is compared to all the identifiers locally stored, and the password is safely recovered. Thus, both the login and the password are inserted into the corresponding controls of the website, programmatically forcing the button press that allows a user to be authenticated on the web platform. When the cognitive stimulation session ends, the robot asks the elderly user to push an accessible button on the screen. When the button is pushed, the robot autonomously returns to the base.

- Google Cloud backend and frontend: Although most data are locally stored by the robot itself and it does not need to use external resources for navigating or doing natural interaction, a backend and frontend have been developed by using the following Cloud services provided by Google: Google App Engine, Datastore and EndPoints Framework. The front end is a website that allows installers to install the robot in a residence for the first time. The backend makes storing data in the Datastore easy. The information acquired from the users and the environment is named *JUNO context* and it includes personal data, DL models and metrics, and semantic and topological maps, among others. This facilitates the installation of a robot unit in one or another institution by simply downloading a new *JUNO context* and deleting all the local information previously stored for other contexts.

## 3. Results

JUNO project started on 1 January, and it ended on 31 December in 2022. During the process of development, different tests were performed, first in the laboratory and next in the real environment.

For testing the system in the laboratory, two real locations were selected: a real home (with main room, three smaller rooms, and the kitchen), and the first floor of the building where the installations of the Department of Automation, Electrical Engineering and Electronic Technology are located. Such department belongs to the Technical University of Cartagena, in the city of Cartagena, in Spain.

The institution selected for the final tests and deployment is an elderly home located in San Pedro del Pinatar, a village near the city of Cartagena. The full information about this place is not shown in this paper for confidentiality.

### 3.1. Validation of the Navigation System

**Figure 5:**
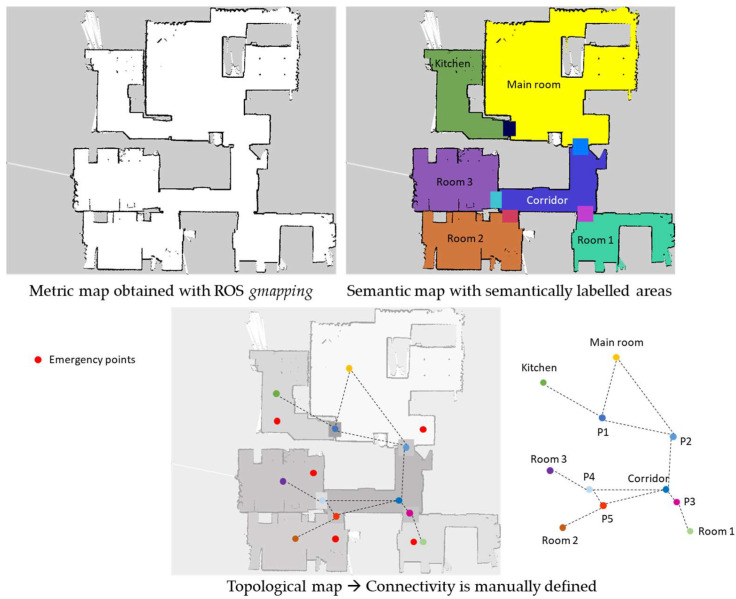
Collection of superposed maps: Metric, semantic and topological with the details of relevant points that represent areas and emergency places. Points with different colors defines representative locations in each specific area of the semantic map. Red points are considered as emergency points and they do not define nodes included in the graph that represents the topological map. Representative points of areas that connect different semantic regions are identified as Pi, where “i” is defined as a number.

For each selected place, a metric map was obtained by using the ROS *gmapping* package, by teleoperating the robot and using the *rviz* ROS utility available in the ROS ecosystem. After saving the map with ROS *map_server*, two files are generated. One of them is an image with extension *.pgm*, which can be easily edited with a typical image processing application, such as GIMP. With this application, a colored map was manually obtained. A different color was assigned to each representative area. Thus, the representation of a semantic map was obtained, since each color is codified by a number, which is used for publishing the semantic map as an occupancy grid message in the topic *semanticmap*. The information that relates each colored area with a semantic label, which describes it, is stored in a file locally saved, which also stores data about the representative points on the map for creating the topological map. Each point also has a semantic label indicating whether the point is the representative position of a specific area or an emergency position. All this information is also stored in the Google Cloud application when a new context is created (see Figure 5).

First, the Navigation stack components, together with the own local planner described in Section 2.3.3, were tested for each map, and several configurations of parameters were used. The *rviz* application was used to define different target goals to prove that the robot was capable of generating safe paths in each real setting and it was able to follow such paths, even in narrow spaces, first without considering dynamic obstacles, because the semantic map was not included in these tests yet.

Then, the semantic and topological maps were included, and the global navigation system was tested again, but using the user interface that allows the use of spoken commands to select the goal. All feasible paths from one place to another were evaluated according to the connectivity defined in the topological map in Figure 5.

**Figure 6:**
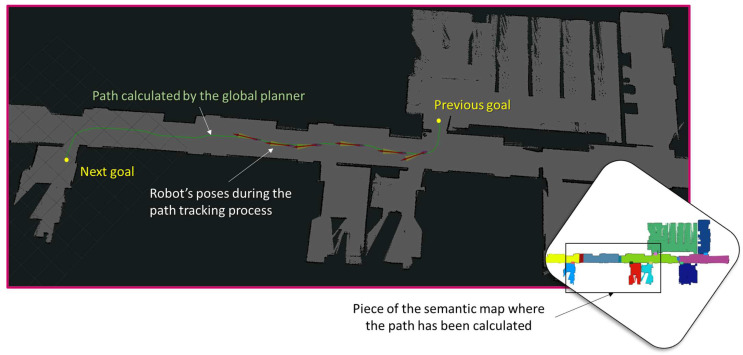
Experiment in the laboratory with a real robot (*rviz*-based view). The path is calculated between two navigation goals, corresponding to two different regions in the semantic path, by the global planner. Arrows represent the different poses of the robot when it is tracking the global path.

**Figure 7:**
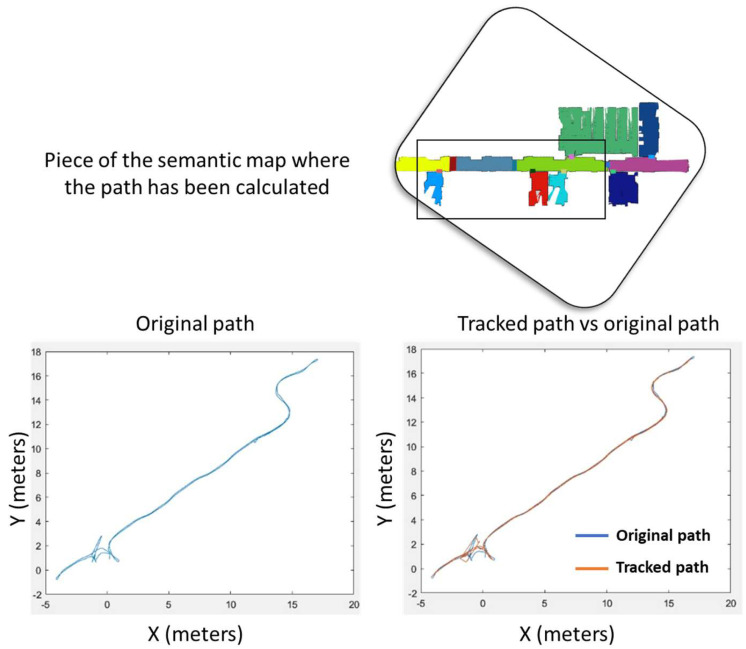
Original and tracked path when the robot is sent to different goal positions during an experiment in the laboratory.

Figure 6 and Figure 7 show how the global planner and local planner contribute to the robot reaching different goal positions in a safe manner. In particular, the map of the laboratory is used in this experiment, where no dynamic obstacle is present, because the objective of the test is to demonstrate that the path tracker is capable of following the path with high accuracy, since both linear and angular velocity are properly configured according to the characteristics of the path. When the curvature of the path is increased, the robot adapts its behavior, and if necessary, it simply rotates until it reaches the direction of the path. Thus, the robot is capable of traversing narrow spaces, such as doors or corridors, without the risk of collision. Moreover, the configuration of the global path planner generates high inflation over static obstacles, ensuring that the generated path crosses through the center of the open doors and corridors, trying to maintain the obstacles located to the same distance on the right and on the left. Note that the error between the original path and the tracked path (both represented in the map frame) is very low, and this demonstrates that the planner and the AMCL algorithm are working properly.

### 3.2. Validation of the Components Aimed at Natural Human–Robot Interaction

Each specific component has been individually tested. Furthermore, several tests were made to decide which open-source components were more suitable for carrying out the STT, TTS and NLP tasks.

#### 3.2.1. STT System Validation

The comparison between different STT systems is particularly interesting, and it was carried out by considering the Word Error Rate (WER) metric, calculated as:

(1) $$\mathrm{WER}=\frac{S+D+I}{M}$$

where M is the number of words spoken, S are the substitutions (for example, when a word is replaced), D are deletions (a word is omitted from the transcript), and I are insertions (when a not said word is added). The WER does not take into account the reason why errors may happen, but it is a suitable indicator of the reliability of the STT system with noise absence. The robot was tested in very noisy situations, and it is capable of transcribing sentences if the speaker is relatively clearly speaking, and the sentences are short. In fact, the number of transcribed words has been limited by using different thresholds, considering a good one that includes around 15 words.

**Figure 8:**
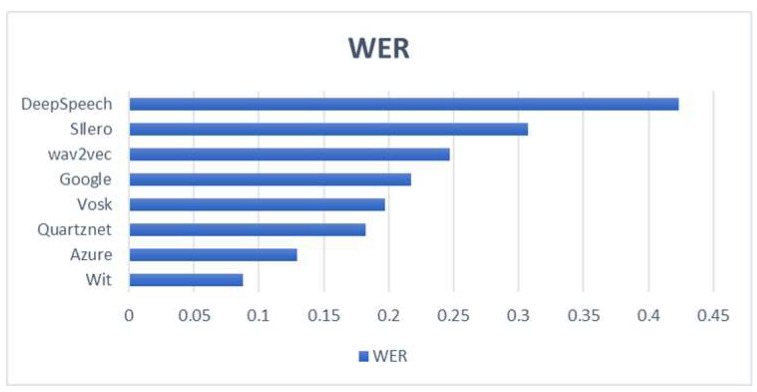
Comparison of accuracy between different STT modules.

Figure 8 shows the WER results for several STT’s by using the Dataset MediaSpeech [69]. The Vosk STT offers a good performance but with low requirements of computational consumption and it can be executed in local mode, without the need to connect to the Internet and without depending on external service providers.

#### 3.2.2. Face Recognition System Validation

As mentioned in Section 2.3.2 and Section 2.3.3, the face recognition module accepts a raw cropped image as a result of applying the face detector component. A reference tensor obtained from *FaceNet* using one image can be stored and then compared to new tensors generated from new images. It is possible to use the Euclidian distance for measuring the differences between values in the tensors (vectors), but it is necessary to empirically define a threshold for recognizing or not a specific user.

However, in this work, several images for each user are used to obtain several tensors. The collection of tensors belonging to a specific person is stored and a label is assigned for identification, for example, a name. Then, it is possible to obtain a collection of tensors as a training dataset with the aim of generating robust classifiers specifically designed for each user. The purpose of the classifier is to differentiate the user from the rest.

During the working process, the robot uses the context downloaded from the Cloud, which contains the information of possible users living in the elderly home. When it is looking for a specific elderly patient to help him/her with the cognitive stimulation session, it applies the model for such a patient to any image acquired by the face detector. If the person is recognized, the exercise is started; otherwise, the robot sends a message to the residence’s worker responsible for it and returns to the base. Consequently, each person has associated his/her own classification model, and then it is necessary to train one for each user. To do this, the tensors for a user labeled with his/her name are used as class 0, and other tensors belonging to other persons are used as class 1. To improve the process of training, data augmentation over original images was done by applying transformations such as “motion,” “reflection” or “color,” among others.

**Figure 9:**
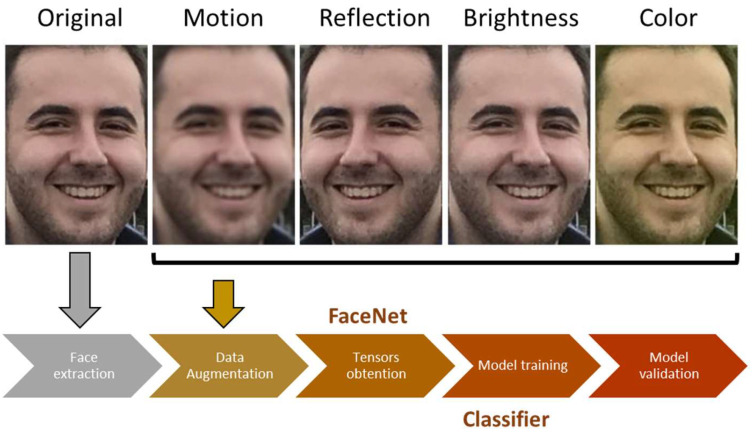
Pipeline for creating each user’s classifier and example of data augmentation.

Figure 9 shows an example of the augmented images used to obtain the classifier of a specific person and the pipeline to obtain it.

Several techniques have been proven for the classification process: A Deep Neural Network (DNN), a Gradient Boosting Decision Tree (GDBT), the K-nearest Neighbors algorithm (KNN) and a Support Vector Machine method (SVM). The Confusion Matrix (CM) has been used for evaluating the performance of each method, since it easily allows several metrics to be calculated: accuracy, precision, recall and F1-score [70].

Each method has its own parameters and they have been heuristically configured. The results that show the performance of each method for two persons (one man and one woman) are as follows:

- Results for the DNN.

**Figure 10:**
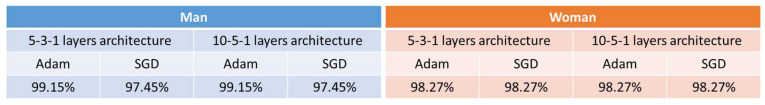
Results of the DNN classifier.

Figure 10 shows the results obtained for the different network structures and optimizers of the net.

- Results for KNN.

**Figure 11:**
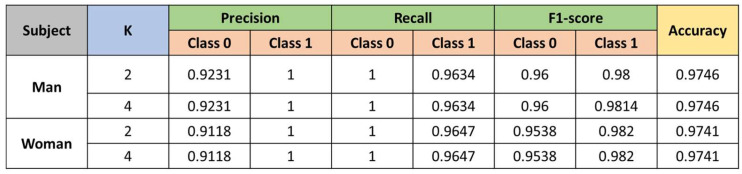
Results of the KNN classifier.

Figure 11 shows the results obtained with the KNN method when K (number of clusters) is configured.

- Results for SVM.

**Figure 12:**
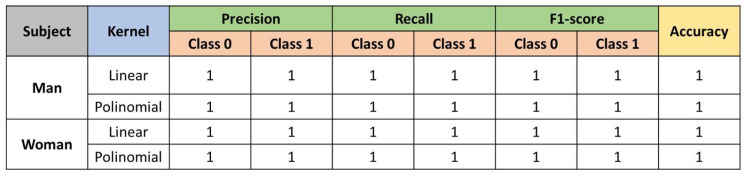
Results of the SVM classifier.

Figure 12 shows the results obtained after testing a lineal kernel and a polynomial one.

- Results for XGBoost.

**Figure 13:**
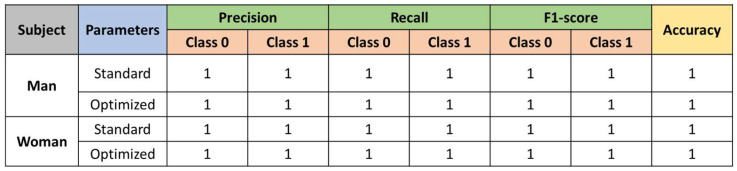
Results of the XGBoost classifier.

Figure 13 shows the results obtained after testing with the standard and optimized parameters.

All the tested methods offer results with high accuracy independently of the configuration of the parameters. The results obtained by all the models are near 100% and the system is capable of recognizing each individual with a high number of true positives and a very reduced number of false positives. The SVM method has been selected for its implementation in the final system because it is computationally efficient, and the precision and recall are very high. In particular, to validate the method, 31 samples of images of the woman were used. The total number of images presented to the model was 116. In this case, all the samples were properly classified. In the case of the man images, 114 images were presented, representing 31 samples of images of the man. All the images were properly classified in this case. These results make sense since FaceNet provides a tensor or vector of features that allows the identification of faces by simply calculating the Euclidian distance. However, using such a distance, the threshold that defines whether one face is or does not belong to a specific person should be empirically calculated, and this procedure is not consistent enough. Therefore, it is better to use a classifier already existing in the state-of-the-art to ensure the robustness of the classification process.

#### 3.2.3. Validation of the NLP Module

**Figure 14:**
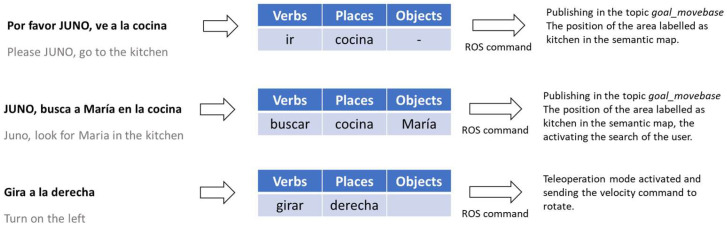
Example of sentences understood by the robot.

To validate the NLP component, the output of the STT module has been analyzed with different sentences that mean motion in Spanish. A simple expert system (combined with the Matcher class described in Section 2.3.2) has been implemented for translating the verbs, places and objects detected in a sentence to the corresponding action for the robot. Figure 14 shows several examples of sentences in Spanish that are properly translated and interpreted.

### 3.3. Validation of the Whole System

**Figure 15:**
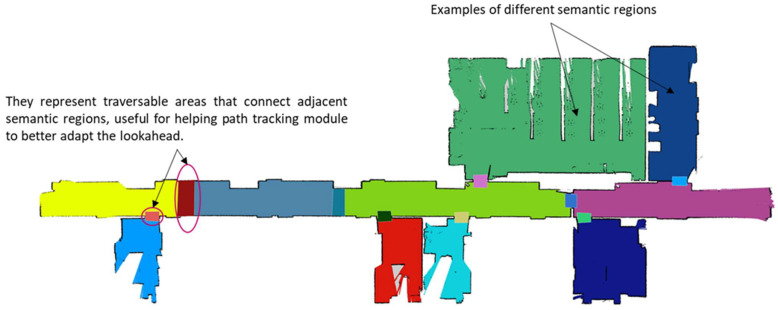
First floor of the department, where several tests have been made.

**Figure 16:**
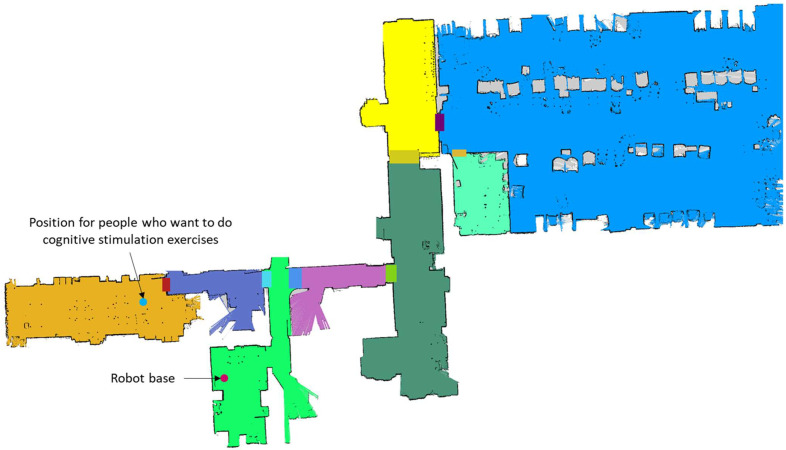
Floor of the elderly residence where the robot has its base and where the stimulation cognitive sessions are applied.

The complete system has been finally validated in three different real environments mentioned at the beginning of Section 3. Two scenarios correspond to laboratory tests and the elderly residence is the setting where the system is currently deployed. The obtained semantic maps (superposed on the metric maps obtained with *gmapping*) are shown in Figure 5 (for the home), in Figure 15 (for the first floor of the department) and in Figure 16 (for the elderly residence).

A wide range of experiments have been conducted at the laboratory level. This has allowed failures to be detected and corrected, mainly those related to tuning the ROS parameters of *amcl* and *move_base* packages. The system has recently been deployed in an elderly institution. By the moment, the residence’s staff is being trained in the use of the robotic platform and navigation tasks are closely supervised by the researchers of the project, manually avoiding dangerous situations by using the teleoperation module if needed.

A number of 12 subjects have been recruited for the final tests. All of them are elderly people, and four suffer early symptoms of cognitive decline. More than half are impaired from a physical point of view, and they need wheelchairs or a walker to go from one place to another. Several of them have auditory and/or vision problems.

From the start of the experiments, all the subjects show a great interest in the robotic platform, and they consider that cognitive stimulation exercises are not only funny, but emotionally healthy.

## 4. Discussion

The proposed system meets the objectives defined at the beginning of the JUNO project (proof of concept). According to the definition of proof of concept, JUNO is a pilot project that demonstrates that the designed robotic platform, together with the software architecture, can be reliably applied in a very complex real setting, such as an elderly residence where vulnerable people live.

The ROS ecosystem provides many facilities for developing robotic software, not only in a research scenario but in a real one, where robots need to be deployed, facing non controllable situations.

The combination of several modules for autonomous navigation, putting together metric, topological and sematic maps, allows the robot to be controlled very easily by simply using spoken commands. A set of recovery behaviors has been specifically designed for this project, mainly related to local planning and how the robot should act when it is not possible to track a trajectory calculated by the global planner.

**Figure 17:**
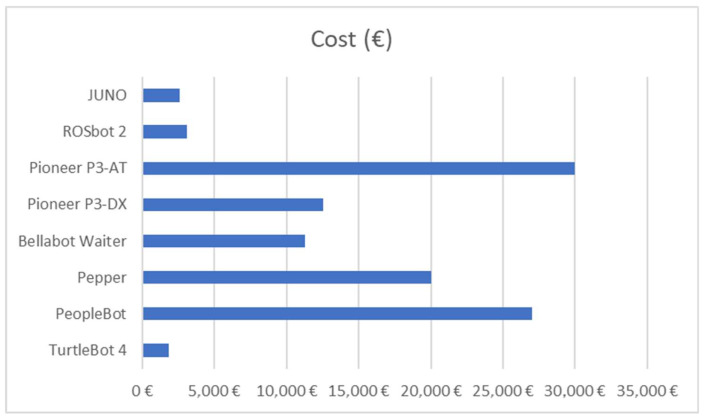
Comparison between different models or commercial robots and JUNO.

One of the main advantages of the system is its low economic cost in comparison to other robots (with similar equipment) found in the market (see Figure 17).

JUNO is considerably cheaper, but its size, dimensions and shape have been consensual by a team of experts that include, not only technicians, but also health professionals, with great expertise in the field of aging research. Furthermore, the behavior of the robot is stable in terms of control and autonomous navigation and comparable to other much more expensive robotic platforms. Finally, most components are capable of working without connecting to the Internet by locally storing results. Such results can be synchronized with the Cloud through the Google Cloud application.

The use of local modules for face detection and recognition provides the ability to carry out tasks in scenarios where the Internet connection is weak or even absent in some moments. Moreover, latency issues are minimized when these kinds of components are locally executed. Note that the selected models for STT, for example, are those used in lightweight devices, reducing computational needs.

The proof of concept has been useful for identifying a set of drawbacks or limitations of the proposed system, which will be addressed in the future through a new project named JUNO+, led by several authors of this work. Such limitations are as follows:

- Although the ROS framework helps developers implement and combine different robotic software packages in a decoupled and distributed manner, it is necessary to create an Intranet where the ROS master runs under a specific IP. In this work, this is not a problem because the system has been deployed in the residence by developers. However, if the installation procedure is expected to be automatized, it is necessary that all the hardware components connect to the same Intranet automatically. The authors are currently working on this issue, and a possible solution is to share such IP through the Google Cloud application, which allows the context of the robot to be saved.
- The semantic and topological maps are currently defined manually, but it would be desirable that installers define the set of topological points while they are teleoperating the system during the installation step by using spoken commands. The authors are also working on tunning an exploration algorithm that allows the robot to build the first map without the need to be teleoperated.
- In the context of an elderly institution where many elderly users are physically impaired and are often helped by sticks, walkers or wheelchairs, and they are susceptible to suffering falls and serious health problems if they are injured, the local planner should be redesigned by considering these issues. In fact, the authors are also currently developing a new local planner capable of allowing the robot to navigate, considering the social protocol and evaluating the dynamic objects that appear on its path. To do this, the RGB-D sensor will be used together with the implemented face detector, and from the face, the system will detect the rest of the body of humans moving in the near path of the robot. According to the degree of frailty detected by analyzing the objects surrounding the person (sticks, walkers or similar), the local map will be updated. The robot can only go near a person if there is enough distance to ensure that it is not possible to cause injury to the user.

## Figures and Tables

**Table 1:** List of components for the JUNO robotic platform, with its economic cost.

Hardware Component	Cost (€)
Touch Screen 13.3”	178.00
Orbbec Astra RGB-D Camera	216.59
RPLIDAR S2 360° Laser Scanner	399.00
MSI Cubi N JSL-033BEU Intel Celeron N4500 computer	210.00
USB 3.0 4 ports HUB	25.00
Two Raspberry Pi Pico microcontrollers	19.00
Lithium-ion 24 V 12 AH battery	135.00
Small consumable electronic equipment	50.00
JUNO differential traction system	266.00
Small consumable mechanical equipment	50.00
JUNO robot structure	1000
Total	2548.59

## Data Availability

Not applicable.
